# Complete mitochondrial genomes of *Nanorana taihangnica* and *N. yunnanensis* (Anura: Dicroglossidae) with novel gene arrangements and phylogenetic relationship of Dicroglossidae

**DOI:** 10.1186/s12862-018-1140-2

**Published:** 2018-02-27

**Authors:** Jia-Yong Zhang, Le-Ping Zhang, Dan-Na Yu, Kenneth B. Storey, Rong-Quan Zheng

**Affiliations:** 1 Key lab of wildlife biotechnology, conservation and utilization of Zhejiang Province, Zhejiang Normal University, Jinhua, Zhejiang Province 321004 China; 2 College of Chemistry and Life Science, Zhejiang Normal University, Jinhua, Zhejiang Province 321004 China; 3 Department of Biology, Carleton University, Ottawa, Ontario Canada; 4 Xingzhi College, Zhejiang Normal University, Jinhua, Zhejiang Province 321004 China

**Keywords:** Dicroglossidae, *Feirana*, Mitochondrial genome, Gene rearrangement, Phylogeny

## Abstract

**Background:**

Complete mitochondrial (mt) genomes have been used extensively to test hypotheses about microevolution and to study population structure, phylogeography, and phylogenetic relationships of Anura at various taxonomic levels. Large-scale mt genomic reorganizations have been observed among many fork-tongued frogs (family Dicroglossidae). The relationships among Dicroglossidae and validation of the genus *Feirana* are still problematic. Hence, we sequenced the complete mt genomes of *Nanorana taihangnica* (=*F. taihangnica*) and *N. yunnanensis* as well as partial mt genomes of six *Quasipaa* species (dicroglossid taxa), two *Odorrana* and two *Amolops* species (Ranidae), and one *Rhacophorus* species (Rhacophoridae) in order to identify unknown mt gene rearrangements, to investigate the validity of the genus *Feirana*, and to test the phylogenetic relationship of Dicroglossidae*.*

**Results:**

In the mt genome of *N. taihangnica* two *trnM* genes, two *trnP* genes and two control regions were found. In addition, the *trnA*, *trnN*, *trnC*, and *trnQ* genes were translocated from their typical positions. In the mt genome of *N. yunnanensis*, three control regions were found and eight genes (*ND6*, *trnP*, *trnQ*, *trnA*, *trnN*, *trnC*, *trnY* and *trnS* genes) in the L-stand were translocated from their typical position and grouped together. We also found intraspecific rearrangement of the mitochondrial genomes in *N. taihangnica* and *Quasipaa boulengeri*. In phylogenetic trees, the genus *Feirana* nested deeply within the clade of genus *Nanorana,* indicating that the genus *Feirana* may be a synonym to *Nanorana.* Ranidae as a sister clade to Dicroglossidae and the clade of (Ranidae + Dicroglossidae) as a sister clade to (Mantellidae + Rhacophoridae) were well supported in BI analysis but low bootstrap in ML analysis.

**Conclusions:**

We found that the gene arrangements of *N. taihangnica* and *N. yunnanensis* differed from other published dicroglossid mt genomes. The gene arrangements in *N. taihangnica* and *N. yunnanensis* could be explained by the Tandem Duplication and Random Loss (TDRL) and the Dimer-Mitogenome and Non-Random Loss (DMNR) models, respectively. The invalidation of the genus *Feirana* is supported in this study.

## Background

Vertebrate mitochondrial (mt) genomes are closed circular molecules that generally have lengths varying from 15 to 27 kb [1]. They typically encode 37 genes including two ribosomal RNAs (12S and 16S rRNAs), 22 transfer RNAs (tRNAs), 13 protein–coding genes, and one long non-coding region (NCR) called the control region (CR; also referred to as the D–loop region) [2, 3]. The mt genome has several valuable characteristics including small size, rapid evolutionary rate, relatively conserved gene content and organization, maternal inheritance, and limited recombination [4]. Complete mt genomes have been extensively used to test hypotheses about microevolution, to study population structure, phylogeography, and phylogenetic relationships at various taxonomic levels, and to identify cryptic species [2, 5, 6]. The mitochondrial DNA (mtDNA) of many neobatrachian anurans shows gene rearrangement of the relative position of *NADH dehydrogenase* subunit 5 (*ND5*); this has been reported in Ranidae, Dicroglossidae, Mantellidae and Rhacophoridae [7–11]. Rearrangements of two *transfer ribonucleic acid M* (*trnM*) genes were also reported in dicroglossid and mantellid mt genomes [5, 8–10, 12–17]. Other mt genomic rearrangements can also be found in some species of neobatrachians. For example, *Rhacophorus schlegelii* [11], *Mantella madagascariensis* [9], and *Rana kunyuensis* [17] possessed duplicated control regions. By contrast, *Nanorana taihangnica* [13] lost the *trnT* gene and *Polypedates megacephalus* [18] lost the *ATPase subunit 8* (*ATP8*) and *ND5* genes. Gene rearrangements in the mitochondrial genome can be mainly explained by six available models: the recombination model [8, 19], the Tandem Duplication and Random Loss model (TDRL) [20], the Tandem Duplication and Non-Random Loss model (TDNL) [21], the tRNA miss-priming model [22], the Dimer-Mitogenome and Non-Random Loss model (DMNR) [23] and/or the Double Replications and Random Loss model (DRRL) [24].

Within the Neobatrachia, the monophyly of the combined Mantellidae and Rhacophoridae has been generally accepted, but the relationships of Ranidae, Dicroglossidae and (Mantellidae + Rhacophoridae) have been in controversy. The relationship of ((Dicroglossidae + (Ranidae + (Rhacophoridae + Mantellidae)) was supported by Frost et al. [25], Kakehashi et al. [1], Kurabayashi and Sumida [26], Kurabayashi et al. [27], Li et al. [7], Xia et al. [28], Pyron and Wiens [29] and Yuan et al. [30]. However, Chen et al. [31], Ren et al. [32], Zhang et al. [33] and Zhou et al. [12] supported the relationship of ((Ranidae + Dicroglossidae) + (Rhacophoridae + Mantellidae)). Furthermore, the relationships within Dicroglossidae are extremely problematic, and have received much attention. The dicroglossids are divided into two subfamilies and four tribes: Dicroglossinae (Dicroglossini, Limnonectini, and Paini) and Occidozyginae (Occidozygini) with the classification of spiny frogs and non-spiny frogs belonging to the tribe Paini (Dicroglossidae) remaining obscure [25, 34–36]. The taxonomy of this group has been revised numerous times [37–40]. The genus *Feirana* of tribe Paini including three species (*F. taihangnica*, *F. quadranus* and *F. kangxianensis*) is widely distributed in China [36, 41, 42] and was considered to be a synonym to *Nanorana* by Frost et al. [25, 43] and Che et al. [35, 44]. Although Dubois transferred *Feirana* species to the subgenus *Rana* (*Paa*) [38], Fei et al. [39] assigned them to the newly created subgenus *Paa* (*Quadrana*). Dubois [38] and Fei et al. [40] placed the subgenus *Quadrana* as genus *Feirana*. So, the validity of genus *Feirana* is still unknown*.*

Large-scale mt genomic rearrangements in many Dicroglossidae species have been observed. However, complete information on *Nanorana* and *Quasipaa* mt genomes is still lacking except for *Nanorana parkeri* [15], *Nanorana pleskei* [31]*, Quasipaa boulengeri* [16]*, Quasipaa spinosa* [12], *Yerana yei* [14] and *Nanorana taihangnica* [13]. Compared with neobatrachian families, the dicroglossid mt genomes investigated thus far feature differences in gene arrangements, which gave us more chances to discuss the potential reasons for gene rearrangements in the mitochondrial genome.

In the present study, we determined the complete mt genomes of *N. taihangnica* and *N. yunnanensis* as well as the partial mt genomes of six *Quasipaa* species (Dicroglossidae), two *Odorrana* and two *Amolops* species (Ranidae), and one *Rhacophorus* species (Rhacophoridae). In this paper, we follow the system of anuran taxonomy published by Fei et al. [42] and Frost et al. [43] to prevent unnecessary confusion in taxonomy. The data was used to determine unknown mt gene rearrangements, to investigate the validity of the genus *Feirana*, and to test the phylogenetic relationships of Ranidae and Dicroglossidae.

## Methods

### Ethical statement

The thirteen species studied (*N. taihangnica*, *N. yunnanensis*, *Q. boulengeri*, *Q. exilispinosa*, *Q. jiulongensis*, *Q. robertingeri*, *Q. shini*, *Q. verrucospinosa*, *Odorrana livida*, *O. schmackeri*, *Amolops hongkongensis*, *A. wuyiensis*, *Rhacophorus dennysi*) are not protected by the provisions of the laws of People’s Republic of China on the protection of wildlife. Thus, the experiments in this study were performed with toe-clip tissue samples collected from all frog specimens and stored in 100% ethanol. Sample acquisition was reviewed, approved and carried out in accordance with the relevant guidelines of the Committee of Animal Research Ethics of Zhejiang Normal University.

### Sample collection

Specimens included two species of *Nanorana* (*N. taihangnica = F. taihangnica*, *N. yunnanensis*), seven samples belonging to six species of *Quasipaa* (*Q. boulengeri*, *Q. exilispinosa*, *Q. jiulongensis*, *Q. robertingeri*, *Q. shini*, *Q. verrucospinosa*) (Dicroglossidae) including two *Q. boulengeri* samples from two different sites, two species of *Odorrana* (*O. livida*, *O. schmackeri*) (Ranidae), two species of *Amolops* (*A. hongkongensis*, *A. wuyiensis*) (Ranidae), and *R. dennysi* (Rhacophoridae). Information on all of the sequenced samples is shown in Table 1. We were unable to successfully sequence the displacement loop (D-loop) region of these samples except for *N. taihangnica* and *N. yunnanensis* because of highly repetitive regions in the D-loop or other unknown reasons despite many optimization efforts; this is similar to the report of Zhang et al. [45].

**Table 1:** Information on the samples used in this study. Specimen sources and GenBank accession numbers are also shown

Species	Collection Locality	Collection Date	Specimen No.	Accession No.
*N. taihangnica*	Luanchuan, Henan	17-July-2010	LGW-LC-001	KF199146
*N. yunnanensis*	Luoping, Yunnan	22-Oct-2010	STJW-LP-001	KF199150
*Q. boulengeri*	Tongshan, Hubei	7-Jun-2009	JFW-TS-002	KF199152
*Q. exilispinosa*	Wuyishan, Fujian	8-Jun-2010	XJW-WYS-001	KF199151
*Q. jiulongensis*	Wuyishan, Fujian	8-Jun-2010	JLJW-WYS-002	KF199149
*Q. shini*	Longsheng, Guangxi	10-Oct-2011	XJW-LS-001	KF199148
*Q. verrucospinosa*	Pingbian, Yunnan	7-July-2007	DYJW-PB-003	KF199147
*Q. boulengeri*	Luoping, Yunnan	22-Oct-2010	JFW-LPS-002	KX233867
*Q. robertingeri*	Hejiang, Sichuan	7-July-2012	HJJW-HJ-001	KX233868
*O. livida*	Wenzhou, Zhejiang	7-Aug-2013	DLW-WZ-004	KX233865
*O. schmackeri*	Wenzhou, Zhejiang	7-Aug-2013	HCW-WZ-001	KX233866
*A. hongkongensis*	Wuyishan, Fujian	13-July-2013	DYTW-WYS-001	KX233864
*A. wuyiensis*	Wenzhou, Zhejiang	7-Aug-2013	WYTW-WZ-001	KM282625
*R. dennysi*	Yangjiang, Guangdong	1-Sept-2011	DFSW-YJ-002	KX233869

### PCR and sequencing

Total DNA was extracted from the clipped toe of each frog specimen using a DNeasy Tissue Kit (Qiagen, Germany). We amplified overlapping fragments that covered the entire mt genome of *N. taihangnica* and *N. yunnanensis* by normal PCR and long-and-accurate polymerase chain reaction (LA PCR) methods slightly modified from Yu et al. [5, 46] and Zhang et al. [45]. All PCR procedures were performed using a MyCycler Thermal Cycler (Bio-Rad, Hercules, CA, USA). TaKaRa *Ex-Taq* and *LA-Taq* kits (Takara Biomedical, Dalian, China) were used for the normal and LA-PCR reactions. The resulting PCR fragments were electrophoresed on 1% agarose gels, and all target DNAs were purified from excised pieces of gel using a SanPrep DNA Gel Extraction Kit (Sangon Biotech, Shanghai, China) prior to sequencing. The sequences for each fragment were obtained in an automated DNA sequencer (ABI 3730) from both strands. The long fragments were sequenced using specific primer walking of both strands.

### Sequence assembly and analysis

**Fig. 1:**
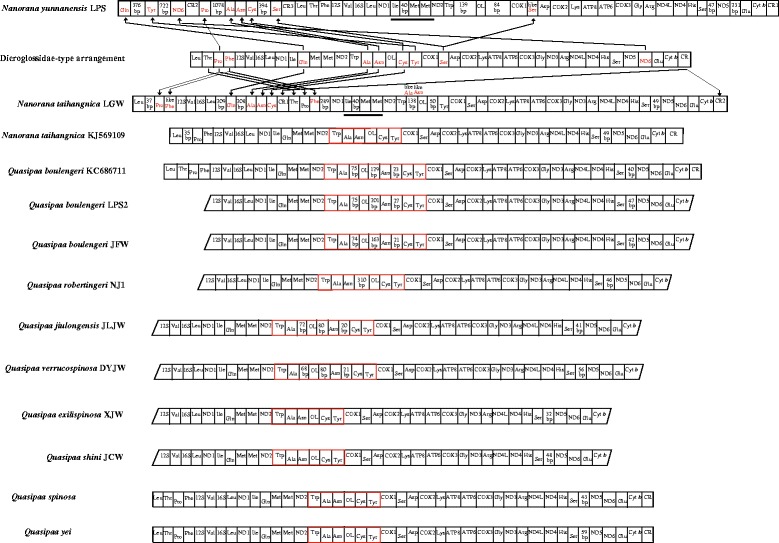
Mitochondrial map of thirteen species of tribe Paini used in this study. The tRNAs are labeled according to the three-letter amino acid codes. The gene name above the median indicates the direction of transcription is from left to right, whereas the gene name below the median indicates right to left. Genes with red letters indicates that the direction of transcription is from right to left. The red square frame shows the region of *WANO*_*L*_*CY* or modified *WANO*_*L*_*CY*

Sequences were checked and assembled using SeqMan (Lasergene version 5.0) [47]. The locations of the 13 protein coding genes and two rRNA genes were determined by comparison with the available RefSeq sequences of closely related anurans downloaded from GenBank using ClustalW in Mega 5.0 [48, 49]. All tRNA genes were identified by their cloverleaf secondary structure using tRNA-scan SE 1.21 [50] or determined by comparison with the homologous sequences of other anurans. The mt genomes (see Fig. 1) of all taxa were analyzed to determine the corresponding mt gene arrangements. The resultant sequences were deposited in GenBank with accession numbers KF199146-KF199152, KX233864-KX233869 and KM282625 (see Table 1).

### Molecular phylogenetic analysis

With the recently increased number of mitochondrial genomes available for Anura, phylogenetic analyses were performed with 83 anurans for which complete or partial mt genomes were available including 14 samples of the 13 species from this study. In total this included the ingroup of 33 species from Ranidae [1, 27, 28, 45, 46, 51–63], 28 species from Dicroglossidae [5, 10, 12–14, 16, 17, 31–33, 64], 13 species from Rhacophoridae [11, 18, 65], one species from Mantellidae [9], one species from Petropedetidae [45], one species from Pyxicephalidae [45], one species from Phrynobatrachidae [45], one species from Ptychadenidae [45], one species from Brevicipitidae (outgroup) [26], one species from Hyperoliidae (outgroup) [26] and two species of Microhylidae (outgroups) [66, 67]. In order to discuss the phylogenetic relationship of Anura, we used the amino acid data and the nucleotides data to compare the identical topology or not according to the methods of Zhang et al. [6] and Zhou et al. [12]. The amino acid sequences of 10 mt protein-coding genes were separately aligned in Mega 5.0 [48] excluding the *ATP8*, *ND5* and *ND6* genes for the following reasons: (a) the *ATP8* sequence was too short in length and had too little good information (only 18 nucleotides or < 0.5% of the total nucleotides of combined PCGs) after G-Block analysis, (b) the loss of the *ND5* gene in some species [18], and (c) the heterogeneous base composition and poor phylogenetic performance for *ND6* which failed to support the consistency analysis with other PCGs [45]. The alignments were revised using Gblocks 0.91b software with the default parameters [68] to select conserved regions of the putative amino acids. We concatenated the alignments of the 10 other mitochondrial protein-coding genes and got an alignment consisting of 2497 amino acid residues as 10Paa dataset. An alignment of 7491 nucleotides sites with 4919 variable informative sites was converted from 2497 amino acids data directly using the amino acid alignment as the backbone. Saturation analysis was performed for subsets with first, second, and third codon positions using DAMBE 4.2.13 [69]. The results showed that the third codon positions were saturated. Thus, we excluded the third codon positions from further phylogenetic analyses and obtained a dataset called 10P consisting of 4994 nucleotide sites from the 1st and 2nd codon positions of the 10 protein-coding genes according to the methods of Cameron et al. [70], Zhang et al. [6] and Zhou et al. [12].

The phylogeny was analyzed using the combined datasets 10P (nucleotides dataset) and 10Paa (amino acid dataset) by the maximum likelihood (ML) and Bayesian inference (BI) methods. To improve the fit of the substitution model to the datasets of 10P and 10Paa, we compared data partitioning schemes according to the Akaike Information Criterion (AIC) and Bayesian Information Criterion (BIC) using the program PartitionFinder v1.0 and PartitionFinderProtein [71]. We set the 10 coding-genes as 20 partitions in dataset 10P and 10 partitions in dataset 10Paa, respectively. For the dataset 10P, twenty-partitions were optimal: 1) first codon positions of the 10 protein-coding genes; 2) second codon positions of the 10 protein-coding genes. The best substitution model of twenty-partitions in ten different genes of dataset 10P is always GTR + I + G. For the dataset 10Paa, ten-partitions were optimal: 10 protein-coding genes with MTMAM. So, the optimal model for 10P with twenty partitions and the optimal model for 10Paa with ten partitions was chosen for ML by the RaxML program [72] and Bayesian analyses by MrBayes3.1.2 [73–75], respectively. ML and BI analyses for datasets of 10P and 10Paa were separately performed using the RaxML program [72] with 1000 bootstrap replications and a modified version of MrBayes3.1.2 [73]. During BI analysis, the following settings were applied: number of Markov chain Monte Carlo (MCMC) generations = 10 million; sampling frequency = 1000; burn-in = 1000. The burn–in size was determined by checking convergences of -log likelihood (−ln L). The robustness of the resulting ML tree was evaluated using bootstrap percentages calculated from nonparametric bootstrap analyses, and statistical support of the resulting BI trees was determined based on Bayesian posterior probability (BPP).

## Results

### Genome organization of mtDNA

**Fig. 2:**
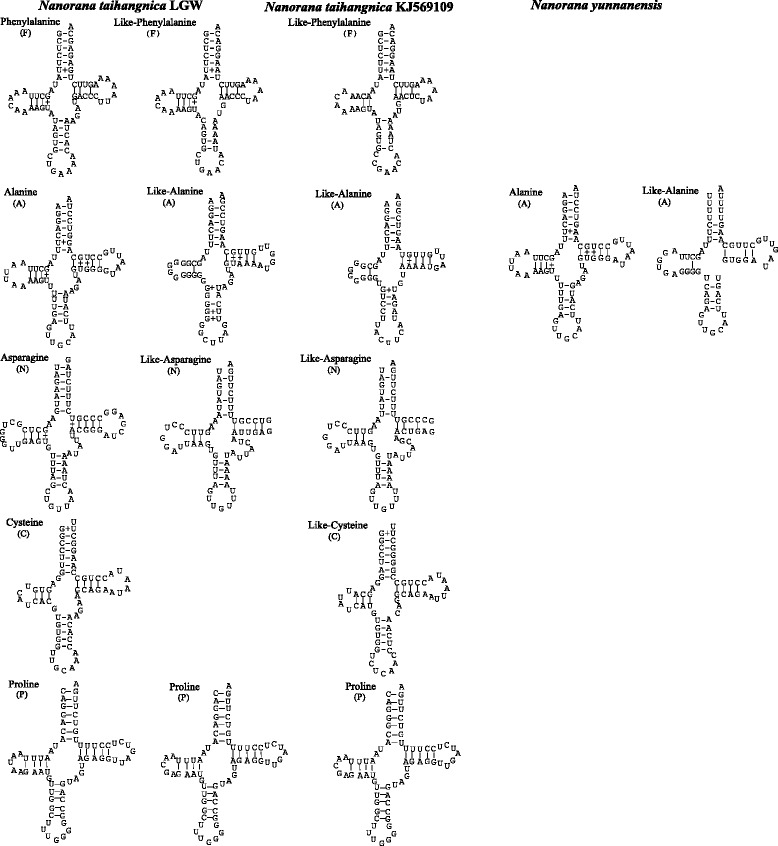
Predicted secondary structures for the tRNA genes and the corresponding pseudo-tRNA genes of *N. taihangnica* and *N. yunnanensis*. Dashes (−) indicate Watson–Crick base pairing and plus (+) indicate G + U base pairing. Arms of the tRNAs (clockwise from the top) are the amino acid acceptor (AA) arm, the TyC (T) arm, the anticodon (AC) arm, and the dihydrouridine (DHU) arm

The *N. taihangnica* mt genome is 21,322 base pairs (bp) in length and contains 13 protein coding genes, two rRNA genes, 24 tRNA genes (including extra *trnM* and *trnP* genes), and 10 NCRs including two control regions (CRs). The two CRs were located between the *cytochrome b* (*Cyt b*) and *trnL* genes (CR1 2014 bp) and between the *trnC* and *trnT* genes (CR2 2698 bp). Remarkably, CR1 and CR2 have nearly identical nucleotide sequences (99.9% similarity with only 1 substitution in 2014 alignment sites) excluding the extra 5′-635 bases and 3′-49 bases in CR2. Tandem duplication of the *trnM* gene and an additional *trnP* gene were found (Fig. 1). The *trnT*-*trnP*-*trnF* tRNA cluster moved from the typical neobatrachian *LTPF* tRNA cluster to a position between the CR1 and *NADH* dehydrogenase subunit 1 (*ND1*) genes. The typical *LTPF* tRNA cluster was replaced by a *trnL*-*trnP*-pseudo *trnF* tRNA cluster. The pseudo-*trnF* showed 89.9% nucleotide similarity with the corresponding *trnF* gene in the *trnT*-*trnP*-*trnF* tRNA cluster. This pseudo-*trnF* contained the same anticodon nucleotides (Fig. 2) compared to *trnF*. The *trnA*, *trnN*, *trnC*, and *trnQ* genes were translocated from their typical positions and replaced by a 40–138 bp NCR (Fig. 1). The *trnQ* gene moved from the typical dicroglossid *IQMM* tRNA cluster to a location between a 209 bp NCR and a 208 bp NCR (Fig. 1) and within the former *IQMM* tRNA cluster the *trnQ* gene was replaced by a 40 bp NCR between the *trnI* and tandem *trnM* genes. The *trnA*, *trnN*, and *trnC* genes also moved from the *WANCY* tRNA cluster to a position between a 208 bp NCR and CR1 (Fig. 1). The positions of the Light-strand replication origin (*O*_*L*_) are located between a 138 bp NCR (non-coding region) and a 52 bp NCR for the translocations of *trnA*, *trnN*, and *trnC* genes, and a *W*-NCR (138 bp)-*O*_*L*_-NCR (52 bp)-*Y* gene cluster was formed in the position of the typical *WANO*_*L*_*CY* gene cluster. Furthermore, a new cluster consisting of a *L*-NCR (209 bp)-*Q*-NCR (208 bp)-*A*-*N*-*C* gene arrangement was observed (Fig. 1). The two *trnP* genes contained the same anticodon nucleotides (Fig. 2). The *trnF* pseudogene contained the same anticodon nucleotides as in *trnF* whereas *trnA* and *trnN* contained different anticodon nucleotides (Fig. 2).

The *N. yunnanensis* mt genome is 23,685 bp in length and contains 13 protein coding genes, two rRNA genes, 23 tRNA genes (including an extra *trnM* gene), and nine non-coding regions (including three control regions) (Fig. 1). Eight genes (*ND6*, *trnP*, *trnQ*, *trnA*, *trnN*, *trnC*, *trnY* and *trnS* genes) in the L-stand were translocated from the typical position to CR regions or near to CR regions and grouped together. CR1, CR2 and CR3 with lengths of 1635 bp, 1581 bp and 1560 bp, respectively, were found between *Cyt b* and *trnQ*, between *ND6* and *trnP*, and between *trnS* and *trnL*, respectively (Fig. 1). The three CRs have a similar sequence with a length of 1372 bp. The typical *WAN(O*_*L*_*)CY* tRNA cluster was replaced by a modified *W*-NCR (139 bp) -*O*_*L*_-NCR (84 bp) arrangement. Through *trnP* translocation, the *LTF* tRNA cluster replaced the *LTPF* tRNA cluster. Through *trnQ* translocation, the *I-*NCR (40 bp)-*MM* tRNA cluster replaced the *IQMM* tRNA cluster. Through *ND6* gene translocation, a 231 bp NCR replaced the *ND6* gene in the original region. Through translocation of the *trnS* gene to between *cytochrome c oxidase subunit I* (*COI*) and *trnD*, the 51 bp NCR replaced *trnS*. A 47 bp NCR was found between the *trnS* and *ND5* genes.

The detailed gene rearrangements of other known dicroglossids, ranids and rhacophorids in this study are described below.

#### *Quasipaa boulengeri*, *Q. jiulongensis*, *Q. verrucospinosa*

The typical *WANO*_*L*_*CY* tRNA cluster was replaced by a *W*-*A*-NCR-*O*_*L*_-NCR-*N*-NCR-*C*-*Y* tRNA cluster, the NCR of which ranged from 20 bp to 201 bp. The typical *IQMM* tRNA cluster was found. A 41–56 bp NCR was also found between the *trnS* and *ND5* genes.

#### *Quasipaa robertingeri*

The typical *WANO*_*L*_*CY* tRNA cluster was replaced by a *W*-*A*-*N*-310 bp NCR-*O*_*L*_-*C*-*Y* tRNA cluster. The typical *IQMM* tRNA cluster was found. A 46 bp NCR was also found between the *trnS* and *ND5* genes.

#### *Quasipaa exilispinosa*, *Q. shini*

The typical *WANO*_*L*_*CY* and *IQMM* tRNA clusters were also found in *Q*. *spinosa* and *Q. yei*. A 32–48 bp NCR was found between the *trnS* and *ND5* genes.

#### *Odorrana livida*

The typical *WANO*_*L*_*CY* tRNA cluster and a 52 bp NCR between the *ND5* and *ND6* gene were found.

#### *Odorrana schmackeri*

The typical *WANO*_*L*_*CY* tRNA cluster was replaced by a *W*-*A*-NCR-*O*_*L*-_NCR-*C*-*Y* tRNA cluster. A 296 bp NCR with tandem sequence between the *ND5* and *ND6* genes was found.

#### *Amolops hongkongensis*, *A. wuyiensis*

The typical *WANO*_*L*_*CY* tRNA cluster and no NCR between the *ND5* and *ND6* gene were found.

#### *Rhacophorus dennysi*

The typical *WANO*_*L*_*CY* tRNA cluster was found and the *ND5* gene between *trnS* and *ND6* was translocated to the region between CR and *trnT*.

### Phylogenetic analysis

**Fig. 3:**
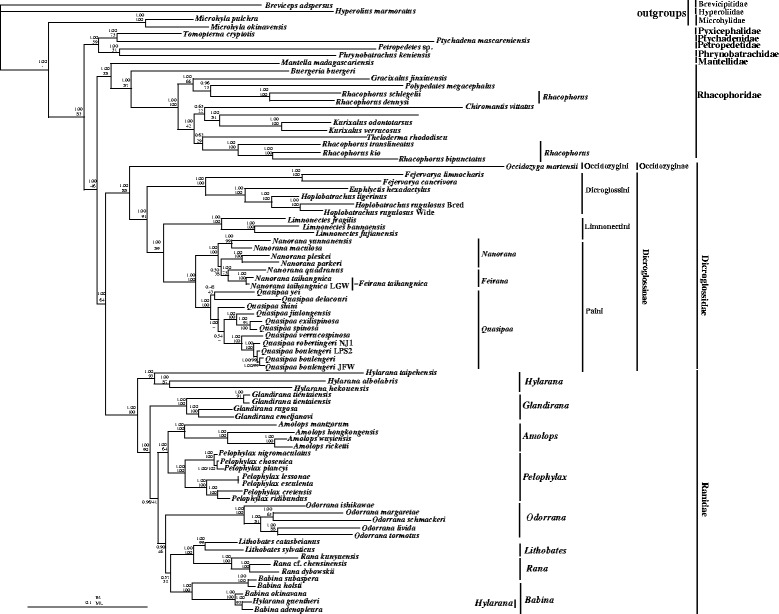
Phylogenetic relationships of Dicroglossidae, Ranidae, Mantellidae and Rhacophoridae based on 10 protein-coding genes using nucleotide datasets. Phylogenetic analyses using nucleotide datasets were carried out for the 83 frogs based on all 10 protein-coding genes from their respective mt genomes. Branch lengths and topology are from the BI analysis. The tree was rooted with four out-groups (*Microhyla pulchra* (NC_024547), *M. okinavensis* (NC_010233), *Breviceps adspersus* (NC_023379) and *Hyperolius marmoratus* (NC_023381)). Numbers above the nodes are the bootstrap values of ML in the bottom and the posterior probabilities of BI in the top

**Fig. 4:**
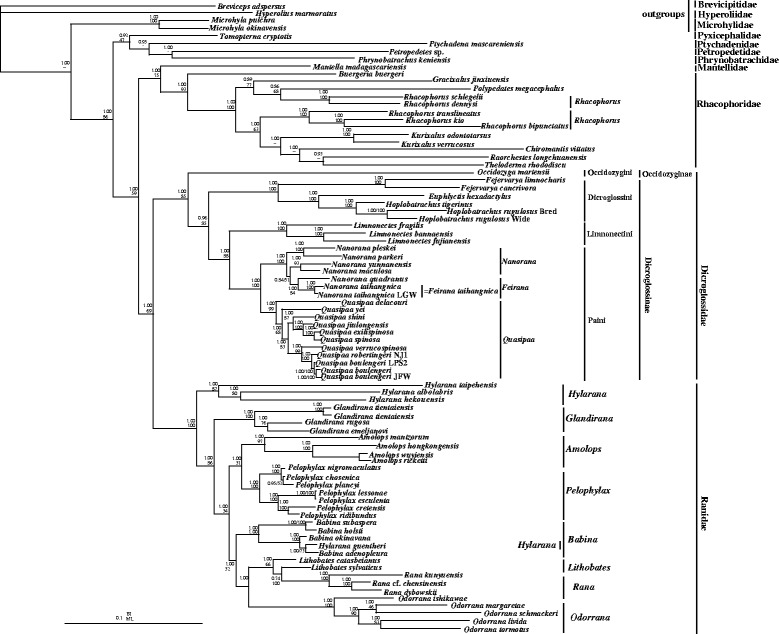
Phylogenetic relationships of Dicroglossidae, Ranidae, Mantellidae and Rhacophoridae based on 10 protein-coding genes using amino acid datasets. Phylogenetic analyses using amino acid datasets were carried out for the 83 frogs based on all 10 protein-coding genes from their respective mt genomes. Branch lengths and topology are from the BI analysis. The tree was rooted with four out-groups (*Microhyla pulchra*, *M. okinavensis*, *Breviceps adspersus* and *Hyperolius marmoratus*). Numbers above the nodes are the bootstrap values of ML in the bottom and the posterior probabilities of BI in the top

All BI and ML phylogenetic analyses performed in this study showed similar topologies (Figs. 3 and 4). In the phylogeny of Dicroglossidae, Ranidae, Mantellidae and Rhacophoridae, the monophyly of Dicroglossidae, Ranidae and Rhacophoridae are well supported. Dicroglossidae is a sister clade of Ranidae (1.00 in posterior probability for nucleotides and amino acids datasets; 64% and 69% bootstrap frequencies for nucleotides and amino acids, respectively), and Mantellidae is a sister clade of Rhacophoridae (1.00 in posterior probability for nucleotides and amino acids datasets; 58% and 75% bootstrap frequencies for nucleotides and amino acids, respectively). Then the clade of (Ranidae + Dicroglossidae) is a sister clade of (Mantellidae + Rhacophoridae) (1.00 in posterior probability for nucleotides and amino acids datasets; 46% and 59% bootstrap frequencies for nucleotides and amino acids, respectively).

In the dicroglossid clade, Dicroglossidae was divided into two clades: Occidozyginae and Dicroglossinae (Figs. 3 and 4). Among the dicroglossid frogs in this study, *O. martensii* (Occidozyginae: Occidozygini) occupied the basal phylogenetic position (1.00 in BI of both datasets; 88% in ML of nucleotide and 85% in ML of amino acids). The monophyly of *Quasipaa* and *Feirana* was supported but the monophyly of *Nanorana* was not supported because the clade of (*N. taihangnica* (*=F. taihangnica*) + *N. quadranus* (*=F. quadranus*)) was supported within the clade of *Nanorana* (Figs. 3 and 4). In *Quasipaa*, the relationship of *Q. delacouri* + (*Q. yei* + ((((*Q. spinosaa* + *Q. exilispinosa*) + *Q. jiulongensis*) + *Q. shini*) + ((*Q. boulengeri* + *Q. robertingeri*) + *Q. verrucospinosa*))) was supported in BI and ML of nucleotide datasets (most nodes: 1.00 posterior probability, > 68% bootstrap frequencies). In the ranid clade, the monophyly of the Genera *Odorrana*, *Pelophylax*, *Amolops* and *Glandirana* was well supported, but the monophyly of *Babina* and *Hylarana* was not supported because *Hylarana guentheri* (KM035413) clustered into the clade of Genus *Babina*. Using the Blast function in NCBI, we found that the mt genome of *Hylarana guentheri* (KM035413) [57] was the most similar to *Babina adenopleura* (DQ283117) [46] with 98% identity, which suggests that *Hylarana guentheri* (KM035413) was misidentified and possibly corresponds to *Babina adeopleura*..

## Discussion

### The mtDNA arrangement

In Dicroglossidae, a 32–85 bp NCR between the *trnS* (AGY) and *ND5* genes was observed in Paini and Limnonectini, while a 7–39 bp NCR between the *trnS* (AGY) and *ND6* genes was observed in Dicroglossini and Occidozygini for the translocation of the *ND5* gene (Fig. 1). The presence of short non-coding sequences among the rearranged genes has also been observed in previous studies [5]. In this study, both *N. yunnanensis* and *N. taihangnica* have a short apomorphic NCR between *trnI* and *trnM*; this was also reported in *N. quadranus* by Zhang et al. [76].

Interspecies tRNA gene rearrangements are well known [11–16], but few were found in the current work. Comparing the tRNA gene rearrangements of mt genomes in all known frogs, we found that the *LTPF* tRNA cluster, the *IQMM* tRNA cluster and the *WANCY* tRNA cluster can easily undergo gene rearrangement, the phenomenon appearing not only in interspecies but also intraspecies comparisons (eg. *N. taihangnica* and *Q. boulengeri*). Comparing two mt genomes of *N. taihangnica* (=*F*. *taihangnica*) between this study and a previously sequenced *N. taihangnica* [13], we found that different gene rearrangements of the *trnP*, *trnF*, *trnQ*, *trnA*, *trnN* and *trnC* genes, the *IQMM* tRNA cluster and the *WANCY* tRNA cluster existed. The *trnT* gene of the *LTPF* tRNA cluster was lost in the previously sequenced *N. taihangnica* [13], whereas the *trnT* gene between CR1 and *trnP* gene was found in *N*. *taihangnica* of this study. The *L-*NCR (35 bp) *-PF* tRNA cluster in the previously sequenced *N. taihangnica* [13] was also found in *N*. *taihangnica* of this study but an extra *TPF* tRNA cluster between CR1 and a 289 bp NCR occurred in *N*. *taihangnica* of this study. The *IQMM* tRNA cluster was found in previously sequenced *N. taihangnica* whereas the *I-*NCR (40 bp)*-MM* tRNA cluster was found in *N*. *taihangnica* of this study because the *trnQ* gene was translocated. The *WANO*_*L*_*CY* tRNA cluster was found in previously sequenced *N. taihangnica* while W- NCR (138 bp)-*O*_*L*_-NCR (52 bp)-*Y* was found in *N*. *taihangnica* of this study because of the translocation of *trnA*, *trnN* and *trnC*. Comparing mt genomes of *Q. boulengeri* between this study and other known sequences [16, 30], we found different gene rearrangements in the *WANO*_*L*_*CY* tRNA cluster as also found by Xia et al. [77]. In species of the Genus *Nanorana* and *Quasipaa*, two types of tRNA clusters (*I*-NCR-*MM or IQMM*, *WANO*_*L*_*CY or WAO*_*L*_*NCY*) were found. Even in the same species, *N. taihangnica* and *Q. boulengeri*, different tRNA clusters were found, which may motivate future discussions on mitochondrial gene arrangements among *Nanorana* and *Quasipaa* species. This suggests that more mt genomes of *Nanorana* and *Quasipaa* species need to be sequenced to further determine how these different gene arrangements formed.

In *N. yunnanensis*, seven tRNA genes (*trnQ*, *trnA*, *trnC*, *trnY*, *trnS*, *trnN* and *trnP*) and the *ND6* gene on the L-stand were translocated into or near to control regions and grouped together. We did not find any other species of Anura where these gene arrangements existed, but in a fish *Crossorhombus azureus* (Pleuronectiformes: Bothidae) [23] seven tRNA genes (*trnQ, trnA, trnC, trnY, trnS1, trnE, trnP*) and the *ND6* gene encoded by the Light-strand (L-strand) were translocated to a position between *trnT* and *trnF*, which is very similar to our results.

### Possible gene rearrangement mechanisms

**Fig. 5:**
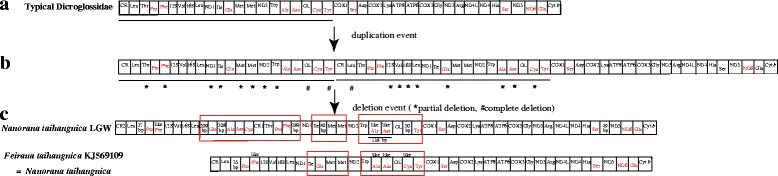
Proposed mechanism of gene rearrangements in *N. taihangnica* under a model of tandem duplication of gene regions and subsequent gene deletions. **a** Typical Dicroglossidae gene order. **b** Tandem duplication in the area from the control region (CR) to *trnY* (*Tyr*) and subsequent deletions of partial genes or complete genes resulting in the derived gene order. **c** State in *N. taihangnica*. * means that the tandem replicated genes were partially deleted and # means that the tandem replicated genes were completely deleted

In *N*. *taihangnica*, we observed several gene rearrangements (extra CR, *trnP* and *trnM* genes as well as translocation of *trnQ*, *trnA*, *trnN*, and *trnC*) in the region between CR and the *cox1* gene. We propose that the gene rearrangements may be explained by the TDRL model. Although long tandem duplication is a very rare event in mtDNA duplication, the duplication can happen between the origin for H-strand replication (*O*_*H*_) in the CR and the origin for L-strand replication (*O*_*L*_) in the *WANCY* tRNA cluster, which is a distance of about two-thirds of the genomic length. The mechanism for duplication between the CR and the *WANO*_*L*_*CY* tRNA genes in *N*. *taihangnica* could be caused by the *O*_*H*_ and *O*_*L*_ structures and be explained by the TDRL model [20], which is similar to the research of Shi et al. [24]. The hypothesized intermediate steps are as follows. Firstly, the above-mentioned *O*_*H*_ and *O*_*L*_ structures initiated DNA synthesis twice during mitochondrial replication, causing tandem duplication of the genes located between the CR and the *WANCY* region in the ancestral mitogenome (Fig. 5). Secondly, one of each of the duplicated gene pairs was randomly deleted completely or partially and then lost its function or became a pseudogene (Fig. 5).

**Fig. 6:**
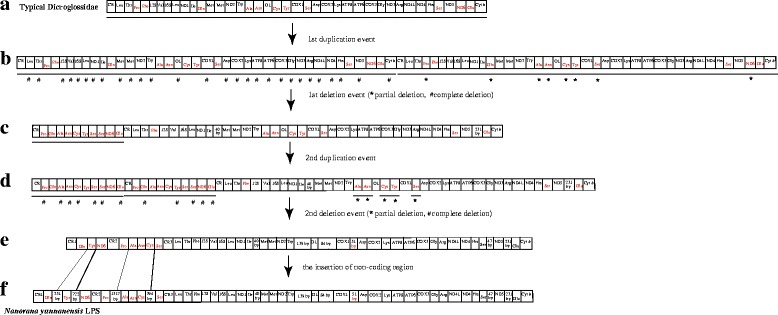
Proposed mechanism of gene rearrangements in the *N. yunnanensis* mitogenome under a model of tandem duplication of gene regions and subsequent gene deletions. **a** Typical Dicroglossidae gene order. **b** The dimeric molecule with two monomers linked head-to-tail and subsequent first deletions of partial genes or complete genes resulting in the derived gene order. **c** State in *N. yunnanensis* after the first duplication and deletion. **d** Tandem duplication in the area from the control region (CR) to *trnE* (*Glu*) and subsequent second deletions of partial genes or complete genes resulting in the derived gene order. **e** State in *N. yunnanensis* after the second duplication and deletion. **f** Tandem duplication in the region of CR1-*trnQ* (*Gln*), CR2-*trnP* (*Pro*) and *trnS* (*Ser*)-CR3, respectively, and subsequent third deletions of partial genes or complete genes resulting in the derived gene order. **g** State in *N. yunnanensis* after the third duplication and deletion. * means that the tandem replicated genes were partially deleted and # means that the tandem replicated genes were completely deleted

In *N. yunnanensis* the genes of the mitogenome are extensively rearranged with clustering of eight genes on the L-strand in the same polarity and three control regions in an unexpected gene order. These special features of eight genes in the same polarity on the L-strand and two noncoding regions were reported in *Crossorhombus azureus* which proposed a new mechanism for gene rearrangement [23]. We can use this gene rearrangement mechanism to explain the polarity of gene rearrangement in *N. yunnanensis.* The hypothesized intermediate steps are as follows. Firstly, the inferred “dimer-mitogenome” intermediate of the *N. yunnanensis* mtDNA (Fig. 6) could be formed by two entire mitogenomes if the two mt genomes were linked by the head-to-tail method. Secondly, some duplicated genes were non-randomly deleted completely except that all ten genes on the L-strand of one mt monomer were retained; some duplicated genes were also non-randomly deleted completely or partially from the other mt monomer (Fig. 6). Thirdly, the region of *CR-trnP-trnQ-trnA-trnN-trnC-trnY-trnS-trnS-ND6-trnE* was duplicated. Fourthly, some duplicated genes were randomly deleted completely. So the Dimer-Mitogenome and Non-Random Loss model (DMNR) [23] and the TDRL model [20] may be more appropriate to explain the gene arrangements in *N. yunnanensis*. But we have no suitable model to explain the four non-coding region (256 bp between trnQ and trnY, 722 bp between trnY and ND6, 1317 bp between trnP and trnA, 394 bp between trnC and trnS).

### Phylogenetic analyses of Dicroglossidae

The evolutionary relationships of dicroglossid taxa indicated by the phylogenetic trees were mostly similar to previously reported molecular phylogeny [5]. Roelants et al. [78] suggested that Occidozygini is a sister clade to ((Dicroglossini + Paini) + Limnonectini), whereas van der Meijden et al. [79] found that *Occidozyga* (Occidozygini) is located within Dicroglossinae. Dubois [37] returned Occidozygini to Dicroglossinae as a tribe based on the strength of evidence produced by van der Meijden et al. [79]. In the present study, Occidozygini was found to be a sister clade to (Dicroglossini + (Paini + Limnonectini)), and Occidozygini (Occidozyginae) was observed to be a basal clade to Dicroglossinae.

In phylogenetic trees, the clade of (*N. quadranus* + ((*N. taihangnica* + *N. taihangnica* (KJ569109)) was clustered into the *Nanorana*. Although *N. taihangnica* and *N. quadranus* belong to the genus *Feirana* according to Fei et al. [42], we draw the conclusion that genus *Feirana* is not valid according to the phylogenetic relationship of *Nanorana* and *Feirana*, which was also supported by Frost et al. [25, 43] and Che et al. [35, 44]

### Invalidation of *Q. robertingeri* as a species

The validity of *Quasipaa robertingeri* is also heatedly debated*.* Che et al. [35] found that *Quasipaa robertingeri* nested deeply within *Q. boulengeri* and suggested that *Q. robertingeri* should be synonymous with *Q. boulengeri*, which is supported by Frost et al. [25]. However, Fei et al. [36, 42] insisted on the validity of *Q. robertingeri* as a species. The data of Pyron and Wiens [29] supported the proposal that *Q. robertingeri* was a sister clade to *Q. shini*, not to *Q. boulengeri*. To compare the genetic divergence we analyzed the complete mt genomes and 16S RNA gene of *Q. boulengeri* and *Q. robertingeri* in Mega 5.0 with the parameter *p*-distance model*.* The average genetic distance between *Q. boulengeri* and *Q. robertingeri* using mt genomes and 16S RNA was determined to be 4.3% and 1.1%, respectively, which is lower than the lowest interspecies mt genomes between *Q. spinosa and Q. exilispinosa* (6.8%) and 16S RNA diversity as a species threshold (3%) [80], respectively. Although *Q. boulengeri* is as a sister clade to *Q. robertingeri* in phylogenetic relationship, the genetic distance between *Q. boulengeri* and *Q. robertingeri* is lower than the genetic distance between interspecies of *Quasipaa.* The different gene arrangement of *Q. boulengeri* and *Q. robertingeri* cannot be used as a species delimitation method because the gene rearrangement can also happened within intraspecies. So we deduce that *Q. robertingeri* may not be a valid species.

## Conclusion

The characteristics of mt genomes and gene arrangements provide novel insights into the phylogenetic relationships among several major lineages of Dicroglossidae. The phylogenetic relationship of ((Ranidae + Dicroglossidae) + (Mantellidae + Rhacophoridae)) is supported in BI analyses. *Feirana* is not a valid genus according to the phylogenetic relationship with *Nanorana*. *Quasipaa robertingeri* may be an invalid species according to genetic divergence. The gene arrangements of *N. taihangnica* and *N. yunnanensis* differed from those of other published dicroglossid mt genomes. The mt genomes are promising markers for discussing the reasons for intraspecies gene rearrangements, and the current results broadens our knowledge of the evolution of anuran mt genomes.

## Abbreviations

AIC: Akaike Information Criterion
*ATP6/8*: *ATPase subunit 6/8*
BI: Bayesian inference
BIC: Bayesian Information Criterion
BPP: Bayesian posterior probability
CR: Control region
*Cyt b*: *cytochrome b*
DMNR: The Dimer-Mitogenome and Non-Random Loss model
DRRL: Double Replications and Random Loss model
MCMC: Markov chain Monte Carlo
NCR: Non-coding region
*ND1–6*: *NADH* dehydrogenase subunit 1–6
rRNAs: Ribosomal ribonucleic acid
TDRL: The Tandem Duplication and Random Loss model
tRNAX: Transfer RNA *acid X*
